# Epidemiological and Clinical Characteristics of the Enterovirus D68 Outbreak in Spain in 2024

**DOI:** 10.1002/jmv.70887

**Published:** 2026-04-04

**Authors:** Juan Camacho, Nerea García‐Ibáñez, María Dolores Fernández‐García, Yasmin Biya, Estrella Ruiz de Pedro, Almudena Gutiérrez, María Carmen Nieto, María Montserrat Ruiz‐García, María Teresa Pastor‐Fajardo, Eduardo Lagarejos, Carlos de Leonardo Simón, Ana Navascués, Antonio Moreno‐Docón, Mónica Gozalo‐Marguello, Carla Berengua, Pedro Antequera, María Dolores Huéscar‐Pascual, Cristina Calvo, María Cabrerizo

**Affiliations:** ^1^ Enterovirus and Viral Gastroenteritis Laboratory, National Centre of Microbiology Instituto de Salud Carlos III Madrid Spain; ^2^ Translational Research Network in Pediatric Infectious Diseases (RITIP) Institute for Health Research IdiPAZ Madrid Spain; ^3^ CIBERESP, Instituto de Salud Carlos III Madrid Spain; ^4^ Hospital U. La Paz Madrid Spain; ^5^ Instituto de Investigación Sanitaria Biobizkaia Hospital Universitario Basurto Bilbao Spain; ^6^ Hospital General U. de Elche Alicante Spain; ^7^ Hospital U. de Gran Canaria Dr. Negrín Las Palmas de Gran Canaria Spain; ^8^ Hospital U. de Navarra Pamplona Spain; ^9^ Hospital Clínico U. Virgen de la Arrixaca Murcia Spain; ^10^ Hospital U. Marqués de Valdecilla Santander Spain; ^11^ Hospital U. de la Santa Creu i Sant Pau Barcelona Spain; ^12^ Hospital U. J.M. Morales Mesenguer Murcia Spain; ^13^ CIBERINFEC Madrid Spain

**Keywords:** enterovirus D68, EV‐D68, outbreak, phylogenetic analysis, respiratory illness

## Abstract

Enterovirus D68 (EV‐D68) is a significant cause of respiratory and neurological disease worldwide. In 2024, Spain experienced its largest recorded EV‐D68 outbreak, accounting for 37.4% of all typed EV (294/892). This study describes the epidemiological, clinical, and phylogenetic features of EV‐D68 infections. Unexpectedly, EV‐D68 infections as it seems were more frequent in adults than in children (57.2% vs. 42.8%, *p* < 0.05), particularly among individuals > 60 years (38.6%). In 84.9% of cases with an EV‐D68 infection, EV‐D68 was the sole pathogen detected. Respiratory pathologies predominated (91.9%), with bronchospasm/wheezing episodes and bronchiolitis in children, and pneumonia and exacerbations of chronic obstructive pulmonary disease in older adults (*p* < 0.05). Older patients showed a more severe clinical profile than pediatric patients, including higher hospitalization rates (79% vs. 59%), longer hospital stays (mean, 10.8 vs. 4.9 days), and more comorbidities (50% vs. 31%) (*p* < 0.01). Phylogenetic analysis revealed two co‐circulating lineages with distinct age‐related tropisms: B3.3 mainly affected children (88.5%), whereas the novel A2/D1.1 infected adults predominantly (79.6%), particularly ≥ 60 years (49.5%) (*p* < 0.05). This pattern may reflect lineage‐specific amino acid substitutions enhancing immune evasion. Neurological disease occurred in only three patients > 60 years infected by the A2/D1.1 lineage, which contained neurovirulent substitutions (I553L, K835E, and T860N). Results support continued genomic and clinical surveillance of EV‐D68.

## 1 Introduction

Enteroviruses (EV) that infect humans belong to the genus *Enterovirus* within the family Picornaviridae. There are more than 116 different types of EV that were once classified into 4 species, *Enterovirus A*, *B*, *C*, and *D* but have been renamed as *Enterovirus alphacoxsackie*, *Enterovirus betacoxsackie*, *Enterovirus coxsackiepol*, and *Enterovirus deconjuncti*, respectively [1, 2]. EV infections are associated with more than 20 clinical syndromes, ranging from benign and short‐lived illnesses (fever, mild respiratory symptoms, rash), to severe and even fatal diseases, such as meningitis and encephalitis, myocarditis, paralysis, and sepsis in neonates and immunocompromised patients. They also cause specific diseases, such as acute hemorrhagic conjunctivitis and hand–foot–mouth disease (HFMD) [3, 4, 5].

Epidemiological surveillance of these viruses is crucial for monitoring their evolution. In recent years, the surveillance of EV infections has demonstrated an increasing public health impact of several non‐polio EV (NPEV) types that have caused major outbreaks worldwide, with a higher number of severe neurological cases. This includes outbreaks of EV‐A71 and EV‐D68 [6]. Except for the three types of poliovirus and, more recently, EV‐A71 and coxackievirus (CV) A16, there are currently no effective vaccines or antiviral therapies against EV infections [7].

Compared to other EV, EV‐D68 is biologically more similar to human rhinoviruses. Until 2014, its prevalence was low, and clinically, it was only associated with respiratory symptoms [8]. However, EV‐D68 emerged in the United States of America (USA) and Canada in 2014 (August–October), where more than 1100 cases were confirmed, mostly in school‐aged children [9]. Acute flaccid myelitis or paralysis (AFM or AFP) complications after EV‐D68 infection were reported [10, 11]. Since then, EV‐D68 outbreaks have been reported in 2016 and 2018 in Europe and other parts of the world, with associated cases of AFM/AFP [12, 13, 14, 15, 16].

The biennial pattern of EV‐D68 circulation was interrupted by the COVID‐19 pandemic, resulting in low case numbers worldwide in 2020. Between the end of 2021 and during 2022, however, EV‐D68 circulated again in Europe and the USA, associated with the emergence of new strains (B3‐derived lineages) and leading to an increase in pediatric hospital admissions for severe respiratory illness [17, 18, 19]. Interestingly, the 2021–2022 resurgence of EV‐D68 was not associated with an increase in AFM cases [19, 20], suggesting a shift in disease outcomes and in the association between EV‐D68 infection and AFM/AFP.

At the phylogenetic level and based on VP1 capsid amino acid analysis, EV‐D68 is divided into four clades: A (Subclades A1 and A2), B (Subclades B1, B2, and B3), C, and D. Clade B appears to have diverged from Clade C in 2007 and the new B1 and B2 subclades were mostly detected in 2014. During the 2016 outbreaks, phylogenetic studies revealed that subclade B3 had replaced the circulating Clades A, B1, and B2. In 2016, China identified the circulation of Clade D, although retrospective analyses revealed D strains circulating as early as 2008; this clade subsequently spread to European countries in 2018. Clade D represents an evolutionary divergence from Subclade A2 and includes two Subclades, D1 and D2 [21, 22, 23, 24, 25].

Since 2014, the Spanish Polio and Enterovirus Laboratory (EL) has conducted continuous surveillance of EV‐D68 circulation in Spain. Previous outbreaks, such as those in 2016 and 2018, as well as the re‐emergence in 2021–2022, were also described by the EL [14, 17, 18, 26]. However, an unprecedented increase in EV‐D68 detections occurred during the autumn of 2024, making it the most significant resurgence since surveillance began. This study had two main objectives: (i) to describe the clinical, phylogenetic, and epidemiological characteristics of EV‐D68 infections identified in 2024, and (ii) to compare these findings with data from previous outbreaks to better understand the recent dynamics of EV‐D68 circulation in Spain.

## 2 Material and Methods

### 2.1 Patients and Clinical Samples

For NPEV surveillance, Spanish hospitals may voluntarily send EV‐positive samples to EL for type characterization at the discretion of clinicians. EL received specimens from EV‐infected patients presenting with various clinical manifestations, primarily respiratory, neurological, or mucocutaneous. During 2024, a total of 892 EV‐positive samples, received from almost 40 hospitals, were confirmed and subsequently tested for typing. More than half of them were respiratory samples (478/892, 53.6%) but also cerebrospinal fluid (CSF) (141/892, 15.8%), blood (138/892, 15.5%), stool (95/892, 10.7%), and cutaneous samples (40/892, 4.5%) were received. Clinical diagnoses were respiratory illnesses (369/892, 41.4%), fever of unknown origin (FUO) (226/892, 25.3%), meningitis, meningoencephalitis or encephalitis (186/892, 20.9%), HFMD (hand, foot and mouth disease) or nonspecific exanthema (83/892, 9.3%), neonatal sepsis (17/892, 1.9%), and others (11/892, 1.2%).

After EV typing, those hospitals with EV‐D68‐positive samples were asked for clinical data on diagnoses of the patients, including descriptions of respiratory or neurological symptoms, coinfections, hospitalizations, intensive care unit (ICU) stays, and comorbidities.

### 2.2 Type Identification and EV‐D68 Whole Genome Sequencing

Genotyping of EV‐positive samples was performed using previously described RT‐nested PCR assays targeting the 3′‐VP1 region of EV Species A, B, C, and D68, followed by Sanger sequencing and BLAST analysis [26, 27]. Viral RNA for genotyping was extracted using the QIAsymphony instrument and the QIAsymphony DSP Virus/Pathogen Midi Kit (Qiagen, Hilden, Germany).

In addition, whole‐genome sequencing (WGS) was performed on selected EV‐D68–positive samples from 2024, based on the sample availability and clinical and epidemiological relevance, prioritizing cases with neurological involvement or more severe clinical presentations (*n* = 9). Viral RNA was extracted using the Quick‐RNA Viral Kit (Zymo Research, Irvine, CA, USA). Sequencing libraries were prepared using the NEBNext Ultra II Directional RNA Library Prep Kit (New England Biolabs, Ipswich, MA, USA), followed by DNA target enrichment using a Twist Bioscience panel (Twist Bioscience, South San Francisco, CA, USA), as previously published protocols [28]. Enriched libraries were sequenced on an Illumina MiSeq platform (2 × 150 bp) (Illumina, San Diego, CA, USA).

### 2.3 Phylogenetic Analysis

EV‐D68 sequences obtained in this study were aligned and analyzed phylogenetically using MEGA12 Software. The analyses were based on the whole genome (7349 bp) or in the second half of the VP1 region, a fragment of 450 bp (2881–3324 nt positions corresponding to the EVD68 Fermon US/1962 sequence, Accession Number AY426531). This 3′‐VP1 region provides sufficient sequence variability for reliable EV typing and subtyping assignment [26, 27, 29]. Analysis included 296 Spanish EV‐D68 sequences detected between 2010 and 2024. Additionally, 97 other sequences available in GenBank from American, Asian, and European countries were added. For the analysis of the whole genomes, 32 strains available in GenBank were included in addition to the 12 Spanish sequences obtained. The phylogenetic trees were constructed using the neighbor‐joining method under the maximum composite likelihood model substitution with 1000 bootstrap replicates.

Genetic distances and nucleotide similarities were calculated using MEGA12 with the compute pairwise distances tool and the maximum composite likelihood model applied to the 3′‐VP1 region. Lineage, subclade, and clade assignments were inferred using approximate nucleotide distance ranges derived from previously published studies [30, 31, 32], rather than fixed universal thresholds. Accordingly, pairwise distances of < 2% (> 98% nucleotide identity) were used to indicate the same lineage; 2%–6% (98%–94%) the same subclade; 6%–10% (94%–90%) the same clade; and 10%–20% (90%–80%) different clades.

### 2.4 Amino Acid Sequence Analysis

Amino acid sequences from the EV‐D68 whole genomes were analyzed in relation to the coding sequence of the Fermon strain and other published sequences to identify amino acid substitutions associated with specific subclades and genetic variability.

Additionally, the obtained sequences were analyzed for the presence of specific amino acid substitutions previously associated with neuropathogenesis, I553L, D554N, A650T and K835E [33], and T860N [34].

### 2.5 Statistical Analysis

Clinical characteristics and laboratory variables were compared using Student's *t*‐test for continuous variables and Fisher's exact test or the *χ* ^2^ test for categorical variables, as appropriate. When comparing more than two groups with non‐normally distributed data, the Kruskal–Wallis test was applied. A two‐sided *p* < 0.05 was considered statistically significant.

## 3 Results

### 3.1 Epidemiological and Clinical Characteristics of EV‐D68 Infections

**Figure 1:**
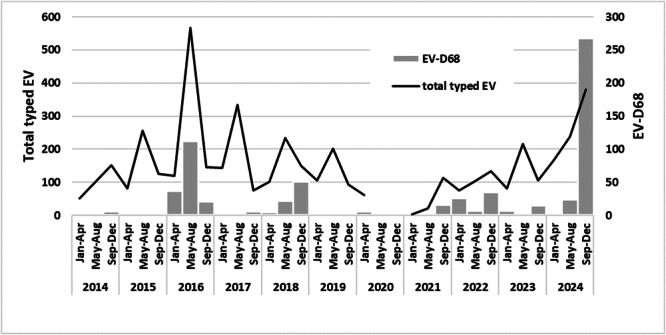
Annual and 4‐month distribution of total EV‐positive samples typed and EV‐D68 identified in the EL between 2014 and 2024.

Of the 892 EV‐positive samples received during 2024, 785 (88.0%) were successfully typed, 563 from children (< 15 years old) and 222 from adults. EV‐D68 accounted for 37.4% (294/785) of all typed enteroviruses and was the most frequently detected type. Furthermore, 2024 was the year with the highest number of EV‐D68 detections since 2014, when the specific surveillance of this EV started in the EL (Figure 1). EV‐D68 was the prevalent EV infection in adults, patients ≥ 15 years of age (76.6%, 170/222 typed EV), whereas in children, patients < 15 years, it was the second most frequent typed EV (22%, 124/563 typed EV) (*p* < 0.0001).

**Figure 2:**
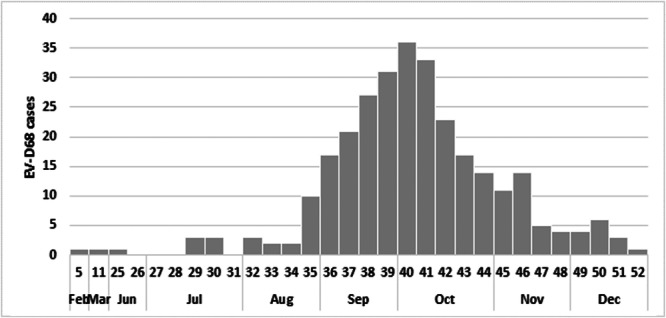
Total EV‐D68 cases per week/month studied in the EL during 2024.

EV‐D68 was detected mainly in the autumn of 2024, with a peak incidence between September and mid‐November (Weeks 35–46), accounting for 83% (244/294) of the total EV‐D68 cases (Figure 2).

**Figure 3:**
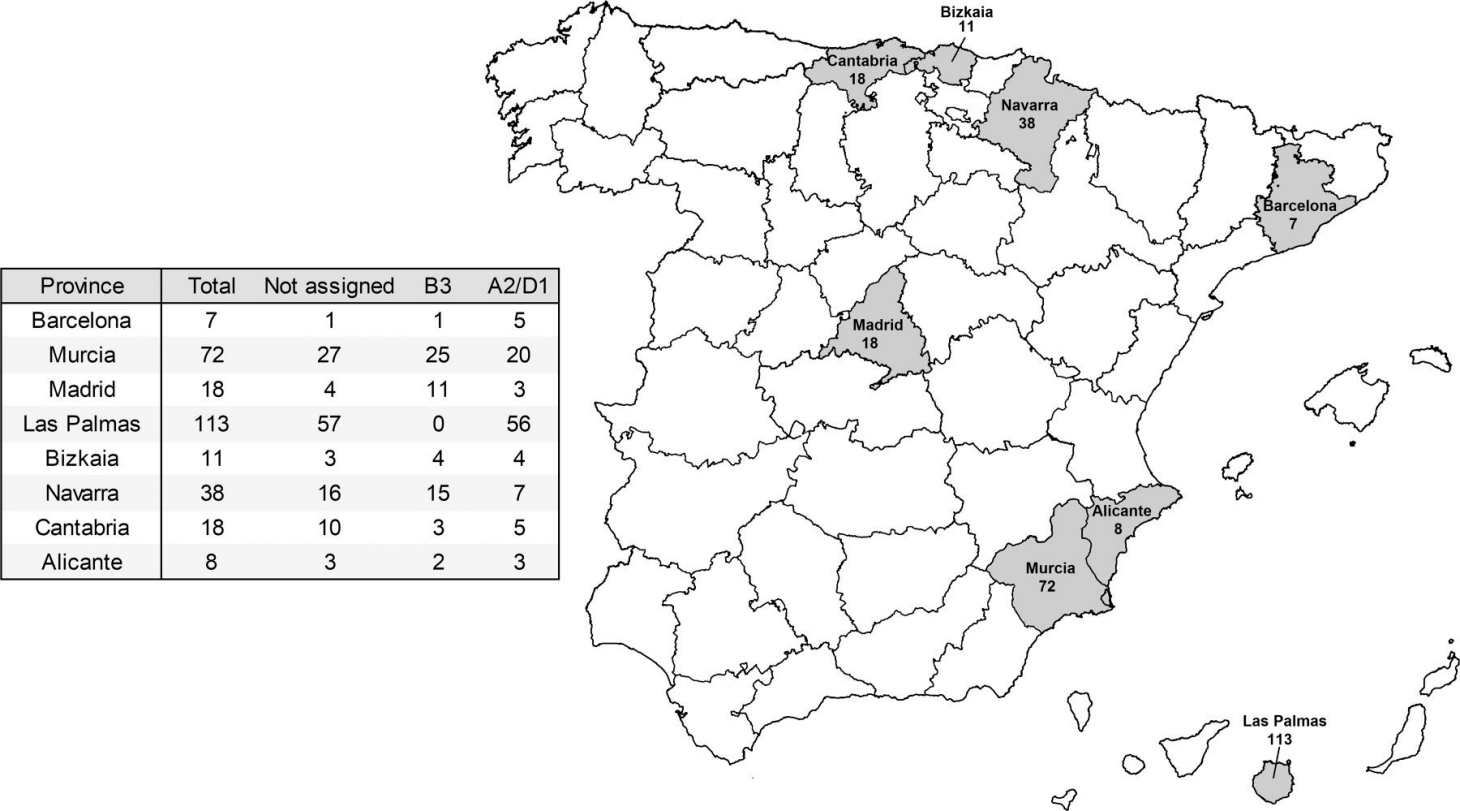
Map showing the Spanish provinces where EV‐D68 infections were reported (in gray) along with the corresponding total number of positive cases. The table details the number of EV‐D68 successfully assigned to a subclade, B3 (*n* = 61) or A2/D1 (*n* = 103), and those not assigned.

EV‐D68–positive samples included 98% throat/nasopharyngeal swabs (288/294), 1.7% five bronchoalveolar lavages (5/294), and 0.3% cerebrospinal fluid (CSF) sample (1/294). One patient provided two separate specimens (a throat swab and a CSF sample). In total, 293 patients were initially included in the study, with clinical data available for 285 patients. These 285 patients were admitted to 8 hospitals located in the Spanish provinces of Madrid, Cantabria, Navarra, Bizkaia, Barcelona, Murcia, Alicante, and Las Palmas. The geographical distribution of 285 EV‐D68‐positive cases is shown in Figure 3.

Among EV‐D68‐infected patients with clinical data, infection was also more frequent in adults than in children (57.2 vs. 42.8%, *p* < 0.001). Specifically, 42.8% (122/285) were < 15 years, 18.7% were adults between 15 and 59 years (53/285), and 38.5% (110/285) were ≥ 60 years (Table 1). Although fewer women than men were affected overall (140 vs. 145, a female‐to‐male ratio of 0.96), the difference was not statistically significant.

**Table 1:** Demographic, clinical, and virological characteristics of 2024 EV‐D68‐infected patients with available clinical information (*n* = 285) stratified by age groups.

Age group	< 15 years (*n* = 122)	15–59 years (*n* = 53)	≥ 60 years (*n* = 110)	*p*
Mean age ± SD, IQR	4.5 ± 4.4, 6	42.2 ± 13.8, 25	76.1 ± 10, 19	
Female/male (ratio)	57/65 (0.9)	24/29 (0.8)	59/51 (1.2)	n.s.
*Diagnosis*				
Respiratory diseases				
URTI (*n* = 88)	33 (27%)	16 (30.2%)	39 (35.5%)	n.s.
Pneumonia (*n* = 32)	3 (2.5%)	11 (20.8%)	18 (16.4%)	**< 0.01**
Bronchospasm/wheezing episode (*n* = 96)	63 (51.6%)	8 (15.1%)	25 (22.7%)	**< 0.01**
COPD exacerbation (*n* = 18)	0	5 (9.4%)	13 (11.8%)	**< 0.05**
Asthma exacerbation (*n* = 16)	6 (4.9%)	4 (7.5%)	6 (5.5%)	n.s
Bronchiolitis (*n* = 12)	10 (8.2%)	1 (1.9%)	1 (0.9%)	**< 0.05**
Nonrespiratory diseases				
FUO (*n* = 9)	4 (3.3%)	3 (5.7%)	2 (1.8%)	n.s.
Sepsis (*n* = 4)	1 (0.8%)	1 (1.9%)	2 (1.8%)	n.s.
Neurological disease (*n* = 3)	0	0	3 (2.7%)	n.s.
Myocarditis (*n* = 3)	0	2 (3.8%)	1 (0.9%)	n.s.
HFMD (*n* = 3)	2 (1.6%)	1 (1.9%)	0	n.s.
Diarrhea (*n* = 1)	0	1 (1.9%)	0	n.s.
*Comorbidities*				
Severe asthma (*n* = 40)	23 (18.9%)	7 (13.2%)	10 (9.1%)	n.s.
Recurrent wheezing (*n* = 12)	9 (7.4%)	1 (1.9%)	2 (1.8%)	n.s.
COPD (*n* = 31)	0	4 (7.5%)	27 (24.5%)	**< 0.01**
Cardiopathies (*n* = 33)	8 (6.6%)	4 (7.5%)	21 (19.1%)	**< 0.01**
Immunosuppression (*n* = 43)	8 (6.6%)	17 (32.1%)	18 (16.4%)	**< 0.01**
Multiples comorbidities (*n* = 10)	1 (0.8%)	0	9 (8.2%)	**< 0.05**
Neurological diseases (*n* = 7)	3 (2.5%)	1 (1.9%)	3 (2.7%)	n.s.
Other comorbidities (*n* = 16)	1 (0.8%)	2 (3.8%)	5 (4.5%)	n.s.
Non‐comorbidities (*n* = 93)	63 (51.6%)	16 (30.2%)	15 (13.6%)	**< 0.01**
*Coinfections*				
Virus (*n* = 33)	21 (17.2%)	6 (11.3%)	6 (5.5%)	**< 0.05**
Rhinovirus (*n* = 24)	16 (13.1%)	4 (7.5%)	4 (3.6%)	**< 0.05**
SARS‐COV‐2 (*n* = 4)	2 (1.6%)	2 (3.8%)	0	n.s.
HPIV (*n* = 3)	3 (2.5%)	0	0	n.s.
RSV (*n* = 2)	0	0	2 (1.8%)	n.s.
Bacteria (*n* = 10)	3 (2.5%)	0	7 (6.4%)	n.s.
Non‐coinfection (*n* = 242)	98 (80.3%)	47 (88.7%)	97 (88.2%)	n.s.
*Hospitalization*				
Hospitalization (*n* = 183)	65 (53.3%)	31 (58.5%)	87 (79.1%)	**< 0.01**
Mean days of hospital stay ± SD, IQR	4.9 ± 5.8, 4	8.7 ± 10.1, 7	10.8 ± 11.2, 8	**< 0.01**
ICU admission (*n* = 15)	5 (4.1%)	6 (11.3%)	4 (3.6%)	n.s.
Mean days of ICU stay ± SD, IQR	5 ± 5.6, 8.5	16.4 ± 16.3, 30	8.5 ± 7.1, 15	n.s.
Death (*n* = 7)	0	0	7 (6.4%)	**< 0.05**
*Phylogenetical analysis*				
Subclade B3 (*n* = 61)	54 (44.3%)	4 (7.5%)	3 (2.7%)	**< 0.01**
Subclade A2/D1 (*n* = 103)	21 (17.2%)	31 (58.5%)	51 (46.4%)	**< 0.01**
Not assigned^a^	47 (38.5%)	18 (34%)	56 (50.9%)	

*Note:* A *p* < 0.05 was considered statistically significant (bold values).

Abbreviations: COPD, chronic obstructive pulmonary disease; FUO, fever of unknown origin; HFMD, hand–foot–mouth disease; HPIV, human parainfluenza virus; ICU, intensive care unit; IQR, interquartile range; n.s., nonsignificant; RSV, respiratory syncytial virus; SARS‐CoV‐2, severe acute respiratory syndrome coronavirus 2; SD, standard deviation; URTI, upper respiratory tract infection.

^a^ Subclade not assigned, due to insufficient coverage and quality of VP1 sequence.

A respiratory disease of variable severity was diagnosed in most cases (91.9%, 262/285). Respiratory illnesses were upper respiratory tract infections (URTI) (33.6%, 88/262) or lower respiratory tract infections (LRTI) (66.4%, 174/262). In patients under 15 years of age, bronchospasm/wheezing episode (51.6%, 63/122) and URTI (27%, 33/122) were the most frequent respiratory presentations, followed by bronchiolitis (8.2%, 10/122). In patients older than 60 years of age, URTI (35.4%, 39/110), bronchospasm/wheezing episode (22.7%, 25/110), pneumonia (16.4%, 14/110), and chronic obstructive pulmonary disease (COPD) exacerbation (11.8%, 13/110) were the prevalent diagnoses. Bronchospasm/wheezing episodes (*p* < 0.01) and bronchiolitis (*p* < 0.05) were statistically more frequent in children than in older patients, while pneumonia and COPD exacerbations were associated with older age (*p* < 0.01) (Table 1).

The most frequent specific URTI symptoms were cough (51.5%, 135/262), rhinorrhoea (23.3%, 61/262), and odynophagia (7.6%, 22/262), while nasal congestion, anosmia, and dysphonia were uncommon (1%–2%). Among LRTI manifestations, dyspnea or acute respiratory distress (69.8%, 183/262), sputum production (24%, 63/262), and wheezing (6.9%, 18/262) predominated. Fever occurred in only 8.0% (21/262) of respiratory patients. Respiratory failure and cough were significantly more frequent in patients < 15 and ≥ 60 years, whereas sputum production was more common in patients ≥ 60 years (*p* < 0.01) (Supporting Information S1: Figure 1).

Among patients with nonrespiratory pathologies (8.1%, 23/285), only three adults aged over 60 years (1%) presented neurological symptoms, including meningoencephalitis (*n* = 2) or stroke (*n* = 1). In one case, EV‐D68 was detected in CSF in addition to the respiratory sample. Other diagnoses such as FUO, cardiopathies, sepsis, HFMD, and diarrhea were also described at lower frequencies (from 0.4% to 3%) but were not significantly associated with a specific age group (Table 1).

The 67.4% (192/285) EV‐D68–positive patients with clinical data had comorbidities. The most frequent respiratory conditions were severe asthma (20.8%, 40/192) and COPD (16.1%, 31/192), while nonrespiratory comorbidities were dominated by secondary immunosuppression (22.9%, 43/192) and cardiopathies (17.2%, 33/192). Less common conditions included recurrent wheezing, neurological disorders, and other systemic diseases. The age comparative analysis of comorbidities revealed that 50% (96/192) of patients with these conditions were over 60 years old, whereas only 31% (60/192) were children < 15 years and 19% (36/192) were adults between 15 and 59 years (*p* < 0.01). Cardiopathies, immunosuppression, COPD, and multiple comorbidities were observed across all age groups. Still, they were significantly more frequent among patients over 60 years, whereas recurrent wheezing was significantly more common in pediatric patients (*p* < 0.05) (Table 1).

Of all patients with EV‐D68 infection, 64.2% (183/285) required hospitalization (mean duration: 8.3 ± 9.8 days, IQR: 7) and 5.3% (15/285) required ICU admission (mean duration: 9.2 ± 11.5 days, IQR: 11). The overall mortality rate was 2.4% (7/285). The mean age of deceased patients was 76.1 ± 12.2 years (IQR: 22), and all had pre‐existing conditions such as cancer, cardiac disease, neurological disorders, or COPD. The age comparative analysis revealed that patients ≥ 60 years old exhibited higher hospitalization rates (0.79, 87/110), in comparison with children (0.53, 65/122) or adults < 60 (0.58, 31/53) (*p* < 0.01), longer stay (*p* < 0.01) and accounted for all recorded fatalities (Table 1). The percentage of patients < 15 years and > 60 years who required ICU admission was similar (5% and 4%, respectively) and lower than that of adults between 15 and 59 years (9%) but the differences were not significant. Adults tended to have longer ICU stays, although the difference was not statistically significant (Table 1).

In 84.9% (242/285) of the samples from patients with available information, EV‐D68 was the only pathogen detected (*p* < 0.01) (Table 1). In 43 patients, coinfections with other viruses were identified: rhinovirus (8.4%, 24/285), SARS‐CoV‐2 (1.4%, 4/285), human parainfluenza virus (HPIV) (1.0%, 3/285), and respiratory syncytial virus (RSV) (< 1%, 2/285). *Mycoplasma pneumoniae*, *Streptococcus pneumoniae*, *Klebsiella pneumoniae*, and *Moraxella catarrhalis* were also detected in the respiratory sample (< 1%, 1/286 each). In addition, *Pseudomonas aeruginosa* (1.0%, 3/285), *Escherichia coli* (< 1%, 2/285), and *Serratia marcescens* (< 1%, 1/285) were identified in the urinary tract of six patients. Viruses, especially rhinovirus, were detected more frequently in pediatric patients compared to adults (*p* < 0.05). Bacterial infections were diagnosed more frequently in patients aged ≥ 60 years compared to children (7 vs. 3), although this difference was not statistically significant (Table 1).

### 3.2 EV‐D68 Phylogenetic Analysis

**Figure 4:**
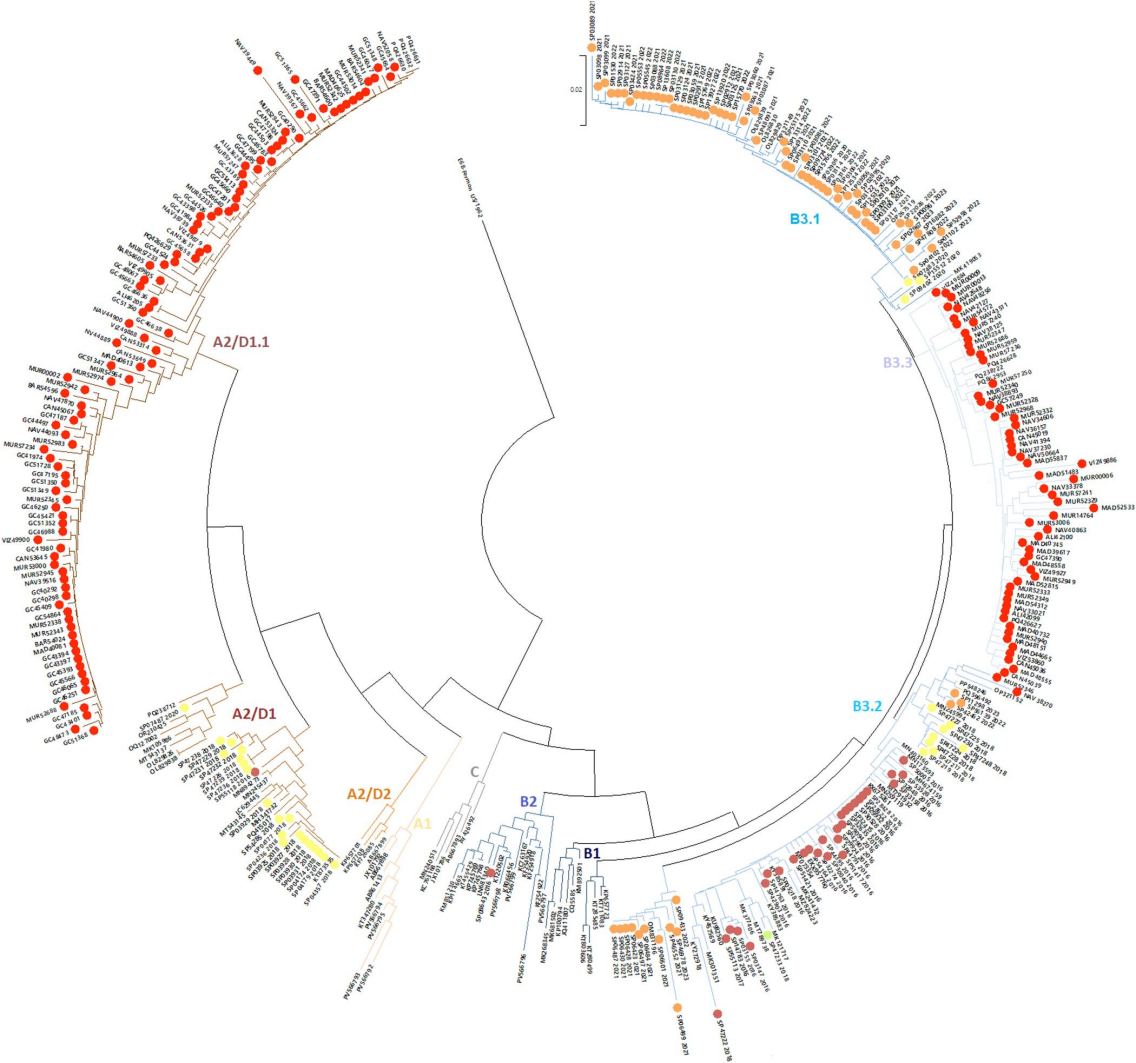
Phylogenetic analysis in 3′‐VP1 region (2881–3324, 450 bp) of EV‐D68 with 164 Spanish sequences detected in 2024, 132 from 2016 and 2023, and 98 strains representative of different clades worldwide, including prototype Fermon US/1962, which was used as the root tree. The tree was constructed in *MEGA12* using the neighbor‐joining method and the maximum composite likelihood model of nucleotide substitutions, with pairwise deletion and 1000 bootstrap replications. The different colors in circles identify the different years of Spanish sequences: 2016–2017 in green, 2018–2020 in yellow, 2021–2023 in orange, and 2024 in red. ID code of the samples from 2024: Spanish region‐sample ID. ID code of the samples from 2016 to 2023: SP‐sample ID‐year. ALI, Alicante; BAR, Barcelona; Can, Cantabria; GC, Gran Canaria (Las Palmas); MAD, Madrid; MUR, Murcia; NAV, Navarra; SP, Spain; VIZ, Bizkaia.

Of all EV‐D68–positive samples typed at the EL during 2024, a total of 164 sequences were selected for phylogenetic analysis based on sequence quality and coverage (Accession Numbers: PV476208–PV476370). Analysis also included 132 Spanish sequences detected between 2016 and 2023 (Accession Numbers: PV566764–PV566907). Phylogenetic tree (Figure 4) and pairwise distance calculations (Supporting Information S4: Table 1) revealed that all EV‐D68 sequences from 2024 clustered into two subclades: B3 and A2/D1. Subclade B3 comprised lineage B3.2 (*n* = 2) and the newly defined lineage B3.3 (*n* = 59), whereas subclade A2/D1 formed a distinct lineage, A2/D1.1 (*n* = 103). Both strains co‐circulated throughout the year (Supporting Information S2: Figure 2).

When comparing the EV‐D68 subclades infecting pediatric and adult patients, almost all sequences belonging to subclade B3 (54/61, 88.5%) were detected in patients younger than 15 years of age. In contrast, 79.6% (82/103) of the sequences belonging to the emerging A2/D1.1 lineage were detected in adult patients, with 49.5% (51/103) infecting individuals older than 60 years of age (*p* < 0.01) (Table 1).

Clinical data were also compared by clade and similar differences to those described between the three age groups were found both in respiratory diagnosis and in the comorbidities presented or hospitalization (Supporting Information S4: Table 1).

Complete EV‐D68 genomes were successfully obtained from six clinical samples collected in 2024 (Accession Numbers: PX970889–PX970894) from patients with neurological (*n* = 2) and respiratory (*n* = 4) diseases. The phylogenetic tree, which included six Spanish sequences from previous years (2010–2021) (Accession Numbers: PV933802–PV933807), showed that five 2024 strains belonged to the A2/D1.1 and one to the B3.3 subclade (Supporting Information S3: Figure 3).

### 3.3 Amino Acid Sequence Analysis

Table 2 shows the relevant amino acid changes observed in the 12 Spanish EV‐D68 genomes (VP2, VP3, VP1, 2B, 2C, 3C, and 3D regions) with respect to the Fermon strain and among several A2/D1 and B3 subclade sequences available in GenBank. Five of these 12 Spanish sequences (202451413, 202454605, 202454600, 202452058, and 202454604) belong to A2/D1.1 lineage and 1 (202449927) belongs to the B3.1 lineage. The remaining 6 sequences belong to A2/D1 subclade (201854285), B3.2 lineage (202206423, 202209433), B3.1 lineage (202009402, 202206493), and A1 subclade (201050324). Clinical presentations and age of the Spanish infected patients were also included.

**Table 2:** Amino acid sequence analysis of EV‐D68, including VP2, VP3, VP1, 2C, and 3D proteins, in nine Spanish sequences (Accession Numbers: PX970889–PX970894 and PV933802–PV933807) and eight reference sequences worldwide, all compared with the Fermon strain.

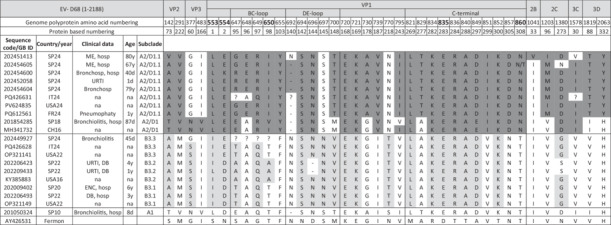

*Note*: The table highlights the main amino acid differences between the newly identified lineages B3.3 and A2/D1.1. The Features column indicates the country and year of detection. A2/D1 substitutions are shaded in dark grey and B3 substitutions are shaded in light grey. Changes previously associated with neurovirulence are highlighted in bold: I553L, D554N, A650T, K835E [33], and T860N [34]. Symbols:?, positions with insufficient sequencing coverage; –, gaps in the sequence.

Abbreviations: Bronchosp, Bronchospasm; CH, China; DB, Difficult breathing; ENC, Encephalitis; FR, France; hosp, Hospitalisation; IT, Italy; ME, Meningoencephalitis; na, not available; SP, Spain; URTI, Upper respiratory tract infection; USA, United States of America.

Analysis of the EV‐D68 coding sequences from the five 2024 whole genomes belonging to subclade A2/D1 revealed more than 100 amino acid substitutions and a single two‐amino acid insertion (p.859_860insRL) compared to the prototype Fermon strain. When compared with other A2/D1 sequences from Italy 2024 (PQ426631), USA (PV624835), and France 2024 (PQ612561), 5–6 amino acid differences were identified, whereas 30–36 differences were observed relative to sequences from China 2016 (MH341732) and Spain 2018 (201854285). Regarding the 2024 B3.3 Spanish complete genome, it showed 80 relevant amino acid substitutions compared to the Fermon strain. When compared with other B3.3 sequences from Italy (PQ426628) and the USA (OP321141), 6 and 8 changes were identified, respectively. There were also differences found with respect to WGS Spanish sequences B3.2 (21 changes) and B3.1 (11 changes).

In the hypervariable region of VP1 (553–860 amino acid numbering), a total of 19 and 10 changes were observed in A2/D1 and B3 sequences, respectively, relative to the Fermon strain. Within the BC loop (641–657 amino acid numbering), five substitutions were identified in A2/D1.1 whole‐genome sequences and 2 in B3.3 (the depth of coverage was not sufficient at certain positions within this fragment; therefore, sequencing of this fragment was only partial). In the DE loop (692–704 amino acid numbering), 3 substitutions and 1 deletion were observed in 4/5 sequences of A2/D1.1, compared to 4 substitutions in B3. Additionally, the C‐terminal region (821–864 amino acid numbering) showed 10 substitutions and 1 insertion in A2/D1 and 7 substitutions in B3 (Table 2). Furthermore, comparison of the 6 EV‐D68 WGS with the 163 3′‐VP1 sequences included in this study showed that the same 11 amino acid substitutions were detected in more than 95% of the Spanish sequences analyzed, and were shared by both A2/D1 and B3 sequences. In addition, five substitutions (G730A, I739V, V795I, A829T, and V851I) were exclusive to A2/D1 (> 95% of cases; *n* = 103), whereas a single substitution (N770T) was restricted to B3 (> 95% of cases; *n* = 61) (Table 2).

Three (I553L, K835E, and I554N) of the five neurovirulent‐associated amino acid changes described previously [33, 34] were found in the five A2/D1.1‐lineage WG sequences obtained, which included those from two patients with neurological pathologies (202451413 and 202454605) (Table 2). However, only one of these mutations was described in the B3.3 Spanish sequence (K835E); another possible mutation, A650T, was in an unresolved sequence fragment, so it could not be confirmed (Table 2). In addition, two other substitutions at positions 554 and 650 were identified: D554E in four A2/D1.1 and in the B3.3 sequences, and A650I in all five A2/D1.1 sequences.

## 4 Discussion

As with other EVs, the epidemiological trends of EV‐D68 exhibit cyclical epidemiological patterns with periodic resurgences, mainly due to waning population immunity and changes in viral evolution. In Spain, major outbreaks were reported in 2016 and 2018, associated with the emergence of subclades B3 and A2/D1, respectively, similar to what happened in many other countries [13, 14, 15, 16, 20, 23, 24, 26]. Subsequently, the EV‐D68 outbreak observed in 2021–2022 coincided with the post–COVID‐19 European upsurge and was related to the emergence of new B3.3 lineages [17, 18, 35]. This study describes a new EV‐D68 outbreak that occurred during the fall and winter of 2024, which represents the largest reported outbreak to date in Spain. EV‐D68 was by far the most frequent EV type detected that year, accounting for 38% of typed EV in the EL.

Phylogenetic analysis revealed that all 2024 strains belonged to two subclades, B3 and A2/D1, with subclade A2/D1 predominating (63% vs. 37%), similar to the Italian study [35] but contrary to what has been described in another 2024 outbreak in Maryland [36]. A2/D1 subclade sequences clustered with those described first in Italy, forming new lineage A2/D1.1 [36], with > 98% intra‐lineage similarity and 2%–6% divergence from A2/D1 sequences detected in 2018.

The B3 subclade, first detected in 2016, has continued to circulate and has evolved into distinct lineages associated with previous outbreaks—Lineage 2 (2016) and Lineage 1 (2021–2022)—both of which co‐circulated in 2023 [16, 17, 18]. Most Spanish B3 sequences (*n* = 59) were grouped within a newly defined lineage (B3.3), also identified in Italy [35], while only two sequences corresponded to lineage B3.2. The predominance of the B3.3 lineage over B3.2 suggests a relative fitness advantage. Supporting this hypothesis, we identified 21 genetic changes between the B3.3 and B3.2 lineages. In virology, such an accumulation of mutations can translate into phenotypic differences—such as improved replication efficiency, immune escape, or enhanced transmissibility—that promote the rapid expansion of certain variants. Although the exact underlying mechanisms and the specific functional impact of these 21 changes remain unclear, it is highly probable that they collectively contribute to the observed fitness advantage of the B3.3 lineage [36]. Regarding seasonality, recent outbreaks (2018, 2021–22, 2024) occurred during colder months, coinciding with other respiratory viruses, while the 2016 outbreak was an exception, peaking in spring [26]. The higher incidence that year may have resulted from a larger susceptible population since previous laboratory data indicated that circulation of this virus had been low until 2015. Ongoing seroprevalence studies in our laboratory aim to clarify these dynamics. Historically, EV‐D68 has predominantly affected the pediatric population, with limited impact on adults [8, 9, 10, 11, 12, 13, 14]. Consistently, recent studies from Europe and the United States reported that only 10%–20% of infections occurred in adults [18, 36, 37, 38], including our previous series [26]. These findings align with seroprevalence studies indicating low antibody levels in children that increase with age, reaching near‐universal seropositivity in adults [39, 40]. In this study, however, 57% of the total EV‐D68 infections occurred in adults, with 39% of them being > 60 years of age. Furthermore, considering the total number of EV infections in children and adults, EV‐D68 was the predominant type among adults (77%), whereas it infected only 22% of pediatric patients. This shift may reflect the antigenic divergence of newer subclades from historical strains or an increased diagnostic detection in this age group due to the inclusion of EV‐D68 assays in multiplex respiratory panels implemented after the COVID‐19 pandemic in Spain. We observed a clear association between EV‐D68 subclade A2/D1 and the adult population; other studies had also reported this [24, 26, 35, 36, 37, 40], but not with such a high mean age (76 years). Adults may lack robust immunity to these emerging variants, whereas prior exposure to older EV‐D68 strains could confer partial protection against subclade B3 [39, 41]. Indeed, subclade B3.3 remains significantly predominant in the pediatric population (89%).

EV‐D68 was the sole pathogen in almost 85% of cases, confirming its etiological role. Viral coinfections predominated among pediatric patients, particularly rhinovirus.

Overall, there were more infected men than women, which is consistent with most reports on EV infections. However, in patients > 60 years of age, the ratio is reversed, probably due to the current higher life expectancy of women compared to men in Spain.

Clinically, EV‐D68 infections were predominantly associated with respiratory pathologies (92%), bronchospasm/wheezing episode, and bronchiolitis being prevalent in children, while in patients over 60 years, pneumonia and COPD exacerbation were more frequent.

Nearly 68% of EV‐D68 infections were in individuals with comorbidities, which may have played a role in the severity of infections. Lower rates of comorbidities were reported in other studies [13, 16, 26], probably due to the older age of the patients in the present study. In fact, half of all infected patients with comorbidities were older than 60 years, with a higher percentage of hospitalization and twice as many days of hospital stay as pediatric patients. Mortality was 2%, occurring exclusively in elderly patients with severe comorbidities. These findings suggest that while EV‐D68 can aggravate underlying conditions, its intrinsic severity may not exceed that of other respiratory viruses, consistent with previous observations [26]. Regarding neurological manifestations, only three patients had a diagnosis of meningoencephalitis or stroke, representing 1% of EV‐D68‐infected patients with available clinical information. No AFM/AFP cases were associated with EV‐D68 infection in 2024. These results contrast with pre‐2020 outbreaks, which found a higher neurological association with EV‐D68 infections [10, 11, 12, 13, 14, 15, 26, 42], but align with post‐pandemic data [17, 18, 19, 20, 35, 36, 37, 38]. In one case of meningoencephalitis, EV‐D68 was detected in both respiratory and CSF samples, supporting its neuroinvasive potential.

Complete genomes from 2024 were obtained in respiratory samples from the two patients with meningoencephalitis and four with respiratory diseases. A comparative analysis of amino acid substitutions was performed to explore the neuroinvasive capacity of the emerged EV‐D68 strains. Most of the sequences, regardless of lineage, had D554E substitution, which had been described recently in B3‐derived lineages associated with a reduced capacity to produce neurological pathologies such as AFP or AFM [38]. However, WGS Spanish A2/D1.1 sequences presented three amino acid substitutions previously associated with a neuropathogenicity phenotype (I553L, K835E, and T860N) [33, 34], while WGS of lineage B3.3 contained only one of them (K835E). Additionally, a different and nonconservative change (A650I), whose functional role is unknown but it was in a position that has been suggested to be involved in modulating the neurovirulence [33], was identified only in sequences of lineage A2/D1. These findings, together with the detection of EV‐D68 in a CSF sample, would suggest that the new lineage A2/D1.1 potentially has greater neuroinvasive capacity than the B3.3 lineage.

Finally, in the few cases diagnosed as HFMD, diarrhea, or sepsis, no other causative agent was identified; however, the detection of EV‐D68 in a respiratory specimen during an epidemic context does not allow us to conclude that this EV was responsible for these specific pathologies.

Concerning the statistically significant associations between phylogenetic classification and clinical data, the observed differences in respiratory manifestations and other clinical variables are likely to be influenced by the age distribution of patients rather than by intrinsic virological differences between subclades.

Complete EV‐D68 genomes from six EV‐D68–positive samples in 2024 and six from previous years confirmed the 3′‐VP1–based phylogenetic classification and allowed analysis of amino acid changes in the B3 and A2/D1‐derived lineages. Particular attention was given to three key regions of VP1: BC‐loop (amino‐acid residues 89–105), DE‐loop (amino acid residues 140–152), and the C‐terminal region of VP1 (amino acid residues 269–312). The BC and DE loops are major capsid antigenic elements involved in antibody recognition and receptor binding, while the C‐terminal region (Antigenic Site III) interacts with antibody CDRs, stabilizing the capsid–antibody complex. Mutations here are a hotspot for immune evasion and antigenic drift have the potential to alter antigenicity and reduce neutralization by pre‐existing antibodies [43, 44, 45]. Consistent with this, almost twice as many amino acid substitutions were observed in A2/D1.1 compared to the B3 subclade (19 vs. 10) across antigenic regions BC‐loop, DE‐loop, and C‐terminal regions, indicating greater sequence variability in these regions in A2/D1.1. Although no functional assays were performed, changes in immunodominant BC/DE loops and the C‐terminal region could be compatible with altered antigenicity and reduced neutralization by pre‐existing antibodies, suggesting a potential driving factor behind the increased circulation of A2/D1.1 observed, particularly among individuals > 60 years of age. Ongoing seroprevalence assays with different EV‐D68 strains, including those isolated in 2024, may help clarify why this outbreak disproportionately affected the elderly population in Spain. Previous seroprevalence studies conducted in the United Kingdom [46] and the Netherlands [47] have provided evidence of sustained EV‐D68 circulation and high levels of neutralizing antibodies across all age groups, indicating long‐standing population exposure. Notably, these studies showed that EV‐D68 was already circulating extensively before the EV‐D68 outbreak reported in 2014. In this context, the emergence of new EV‐D68 lineages with distinct amino acid changes may be associated with partial immune escape and/or altered pathogenicity, contributing to shifts in age distribution and outbreak dynamics.

In summary, this study describes the major EV‐D68 outbreak that occurred in Spain in 2024 due to the co‐circulation of two emerging lineages, B3.3 and A2/D1.1. Results confirm that the virus is not only continuing to spread but is also evolving rapidly, enabling infection of a broader susceptible population, including older adults. This shift in the epidemiological pattern of EV‐D68 may be associated with reduced immunity and immune escape from emerging strains, especially A2/D1.1, which exhibits high amino acid sequence variability. Although no AFM cases were reported and neurological presentations were rare, the detection of more neurovirulence‐associated mutations in the A2/D1.1 strains than in the B3 strains suggests a greater neuropathogenic potential. Furthermore, EV‐D68 infection in patients with multiple comorbidities may exacerbate an already fragile health status, highlighting the importance of improved diagnosis and surveillance.

## Author Contributions

Conceptualization: María Cabrerizo, Juan Camacho. Planning and supervision of surveillance at EVL: María Cabrerizo. Sampling, laboratory EV diagnosis, and collection of clinical data: Almudena Gutiérrez, María Carmen Nieto, María Montserrat Ruiz‐García, María Teresa Pastor‐Fajardo, Eduardo Lagarejos, Carlos de Leonardo Simón, Ana Navascués, Antonio Moreno‐Docón, Mónica Gozalo‐Marguello, Carla Berengua, Pedro Antequera, María Dolores Huéscar‐Pascual, Cristina Calvo. Genotyping: Nerea García‐Ibáñez and Yasmin Biya. Viral metagenomics: Juan Camacho, Estrella Ruiz de Pedro, María Dolores Fernández‐García. Data analysis: Juan Camacho and María Cabrerizo. Funding acquisition: María Cabrerizo. Writing of original draft: Juan Camacho and María Cabrerizo. Revision and editing: María Cabrerizo, María Dolores Fernández‐García, and Cristina Calvo. All authors read and approved the final version of the manuscript.

## Ethics Statement

The study was based on the routine Molecular Non‐Polio Enterovirus Surveillance Programme based at the National Centre for Microbiology (CNM), so specific ethical approval was not required.

## Conflicts of Interest

The authors declare no conflicts of interest.

## Supporting information


**Figure 1S:** Specific respiratory symptoms in EV‐D68‐infected patients by age cohort.

**Figure 2S:** Temporal distribution of EV‐D68 infections by subclade during 2024 (n=164).

**Figure 3S:** Phylogenetic tree of EV‐D68 whole‐genome sequences (WGS) from 6 2024 Spanish samples (accession numbers: PX970889‐PX970894), 6 Spanish sequences from previous years (2010–2022) (accession numbers: PV933802‐PV933807) and 32 reference sequences retrieved from GenBank, including the prototype strain Fermon (USA, 1962), which was used to root the tree.

**Table 1S:** Demographic, clinical, and virological characteristics of 2024 EV‐D68‐infected patients with available clinical information stratified by lineage classification (n=164). **Table 2S:** Comparison of sequence similarity among samples included in the phylogenetic analysis.

## Data Availability

The aggregated data sets generated and analyzed during the current study are available from the corresponding author upon reasonable request. Nucleotide sequences obtained in this study will be deposited in the GenBank database and made publicly accessible following publication. Additional information, including metadata and analytical details, can be provided by the corresponding author upon request.
