# Supplementary figures and images for: Epidemiological and Clinical Characteristics of the Enterovirus D68 Outbreak in Spain in 2024
Source: J Med Virol. 2026 Apr 4;98(4):e70887. doi: 10.1002/jmv.70887 (PMC13049694; doi:10.1002/jmv.70887)

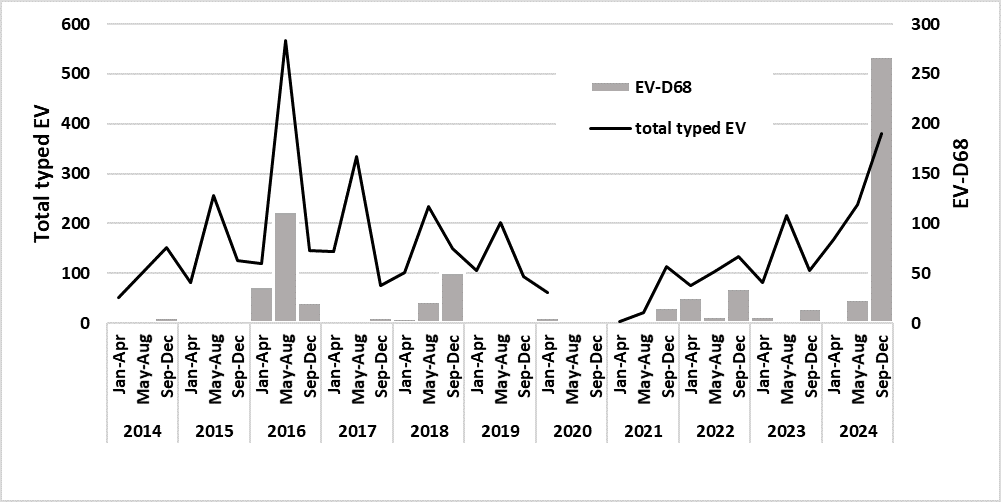

Supplement: Supplementary file 1 — Figure 1S: Specific respiratory symptoms in EV‐D68‐infected patients by age cohort. [file JMV-98-e70887-s003.png]

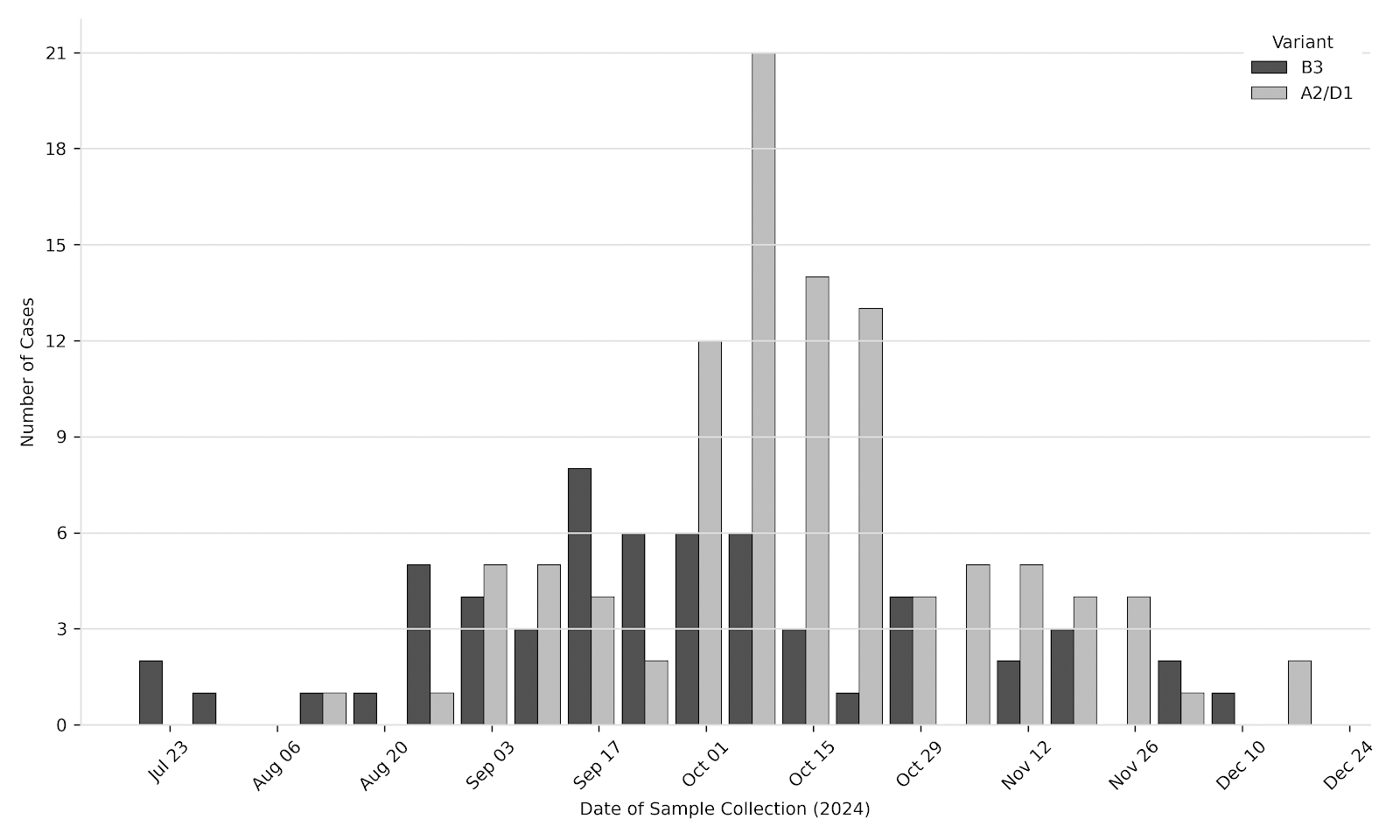

Supplement: Supplementary file 2 — Figure 2S: Temporal distribution of EV‐D68 infections by subclade during 2024 (n=164). [file JMV-98-e70887-s004.png]

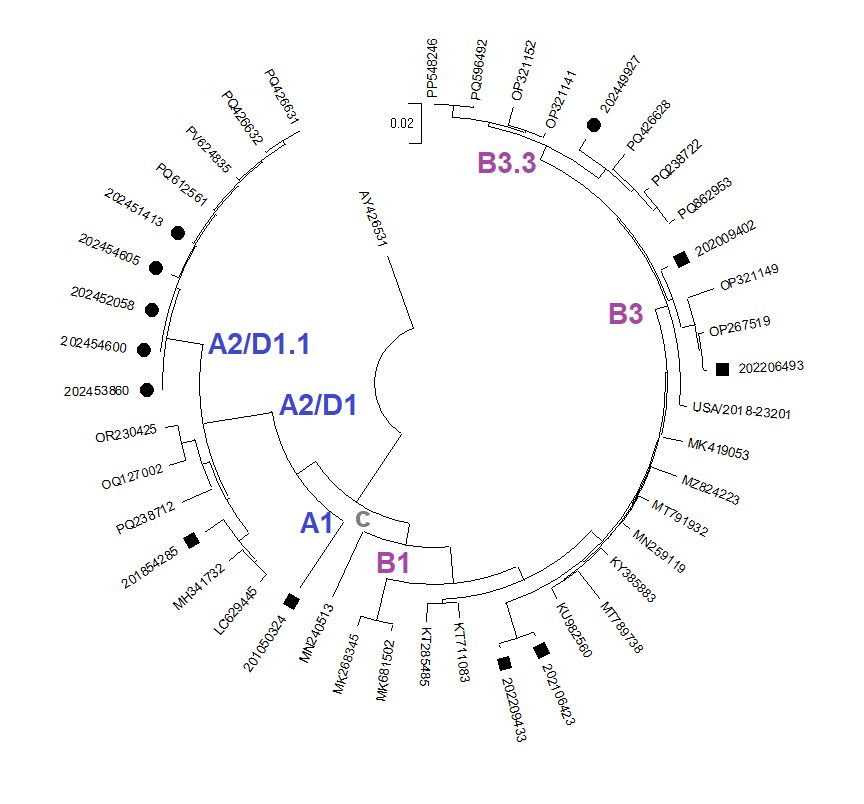

Supplement: Supplementary file 3 — Figure 3S: Phylogenetic tree of EV‐D68 whole‐genome sequences (WGS) from 6 2024 Spanish samples (accession numbers: PX970889‐PX970894), 6 Spanish sequences from previous years (2010–2022) (accession numbers: PV933802‐PV933807) and 32 reference sequences retrieved from GenBank, including the prototype strain Fermon (USA, 1962), which was used to root the tree. [file JMV-98-e70887-s001.png]
