# Supplementary material for: Epidemiological and Clinical Characteristics of the Enterovirus D68 Outbreak in Spain in 2024
Source: J Med Virol. 2026 Apr 4;98(4):e70887. doi: 10.1002/jmv.70887 (PMC13049694; doi:10.1002/jmv.70887)
Supplement: Supplementary file 4 — Table 1S: Demographic, clinical, and virological characteristics of 2024 EV‐D68‐infected patients with available clinical information stratified by lineage classification (n=164). Table 2S: Comparison of sequence similarity among samples included in the phylogenetic analysis. [file JMV-98-e70887-s002.docx]

**Supplementary tables**

**Table 1S.** Demographic, clinical, and virological characteristics of 2024 EV-D68-infected patients with available clinical information stratified by lineage classification (n =164). A p-value <0.05 was considered statistically significant (bold values). Abbreviation: SD, standard deviation; IQR, interquartile range; n.s, non-significant; URTI, upper respiratory tract infection; COPD, chronic obstructive pulmonary disease; FUO, fever of unknown origin; HFMD, hand-foot-mouth disease; SARS-CoV-2, severe acute respiratory syndrome coronavirus 2; HPIV, human parainfluenza virus; RSV, respiratory syncytial virus; ICU, intensive care unit.

| **Subclade classification** | **Subclade B3 (n=61)** | **Subclade A2/D1 (n=103)** | **p-value** |
| --- | --- | --- | --- |
| Mean age ± SD, IQR | 9.8 ± 17.1, 8 | 51.6 ± 30.3, 55 | - |
| Female/male (ratio) | 24/37 (0.65) | 53/50 (1.06) | n.s |
| **Diagnosis** |  |  |  |
| ***Respiratory diseases*** |  |  |  |
| URTI (n=44) | 13 (21.3%) | 31 (30.1%) | n.s |
| Pneumonia (n=19) | 2 (3.3%) | 17 (16.5%) | **<0.01** |
| Bronchospasm/wheezing episode (n=60) | 37 (60.7%) | 23 (22.3%) | **<0.01** |
| COPD exacerbation (n=9) | 0 | 9 (8.7%) | n.s |
| Asthma exacerbation(n=8) | 2 (3.3%) | 6 (5.8%) | n.s |
| Bronchiolitis (n=7) | 4 (6.6%) | 3 (2.9%) | n.s |
| ***Non-respiratory diseases*** |  |  |  |
| FUO (n=6) | 0 | 6 (5.8%) | n.s |
| Sepsis (n=3) | 1 (1.6%) | 2 (1.9%) | n.s |
| Neurological disease (n=2) | 0 | 2 (1.9%) | n.s |
| Myocarditis (n=3) | 0 | 3 (2.9%) | n.s |
| HFMD (n=2) | 2 (3.3%) | 0 | n.s |
| Diarrhoea (n=1) | 0 | 1 (1.0%) | n.s |
| **Comorbidities** |  |  |  |
| Severe asthma (n=23) | 9 (14.8%) | 14 (13.6%) | n.s |
| Recurrent wheezing (n=6) | 5 (8.2%) | 1 (1.0%) | **<0.05** |
| COPD (n=15) | 0 | 15 (14.6%) | **<0.05** |
| Cardiopathies (n=16) | 3 (4.9%) | 13 (12.6%) | n.s |
| Immunosuppression (n=32) | 5 (8.2%) | 27 (26.5%) | **<0.01** |
| Multiples comorbidities (n=5) | 0 | 5 (4.9%) | n.s |
| Neurological diseases (n=5) | 3 (4.9%) | 2 (1.9%) | n.s |
| Other comorbidities (n=9) | 5 (8.2%) | 4 (3.9%) | n.s |
| Non-comorbidities | 31 (50.8%) | 22 (21.4%) | **<0.01** |
| **Coinfections** |  |  |  |
| Virus (n= 18) | 12 (19.7%) | 6 (5.8%) | **<0.01** |
| Rhinovirus (n=13) | 9 (14.8%) | 4 (3.9%) | **<0.05** |
| Bacteria (n=9) | 1 (1.6%) | 8 (7.8%) | n.s |
| Non-coinfection | 39 (63.9%) | 85 (82.5%) | n.s. |
| **Hospitalisation** |  |  |  |
| Hospitalization (n=109) | 37 (60.7%) | 72 (69.9%) | n.s |
| Mean days of hospital stay ± SD, IQR | 4.7 ± 5, 3.5 | 9.7 ± 10, 8 | **<0.01** |
| ICU admission (n=12) | 5 (8.2%) | 7 (6.8%) | n.s |
| Mean days of ICU stay ± SD, IQR | 5 ± 5.6, 8.5 | 14.3 ± 14.4, 17 | n.s |
| Death (n=4) | 0 | 4 (3.9%) | n.s |

**Table 2S*.*** Comparison of sequence similarity among samples included in the phylogenetic analysis. Sequences were grouped according to their phylogenetic clustering into three groups: subclade A2/D1 lineage 1 (A2/D1.1-2024-Spain; n=103), B3 lineage 3 (B3.3-2024-Spain; n=59), and B3 lineage 2 (B3.2-2024-Spain; n=2), and compared both among themselves and with representative sequences from other subclades included in the phylogenetic analysis. The mean ± standard deviation (SD), interquartile range (IQR), and minimum and maximum similarity values (in parentheses) were calculated using the compute pairwise distances in MEGA12.

| % Similarity | A2/D1.1 2024-Spain (n=103) | B3.3 2024-Spain (n=59) | B3.2 2024-Spain (n=2) |
| --- | --- | --- | --- |
| A2/D1.1 2024-Spain (n=103) | 98.0 ± 1.2, 2 (95.2-100) | 85.7 ± 0.9; 1.2 (82.7-88.0) | 86. 1± 1; 1.4 (84.0-88) |
| B3.3 2024-Spain (n=59) | 85.7 ± 0.9; 1.2 (82.7-88.0) | 98.6 ± 0.7; 1.2 (96.1-100) | 96.3 ± 0.5; 0.6 (95.1-98) |
| B3.2 2024-Spain (n=2) | 86. 1± 1; 1.4 (84.0-88) | 96.3 ± 0.5; 0.6 (95.1-98) | 98.0 |
| A2/D1.1 2024-Italy (n=4) | 98.2 ± 1.1; 2 (95.8-100) | 86.3 ± 0.7; 1.1 (84-87.5) | 86.6 ± 0.9; 17 (85.7-87.5) |
| B3.3 2024-Italy (n=2) | 86.2 ± 0.7; 0.8 (84.2-87.5) | 98.5 ± 0.9; 1.2 (95.8-100) | 96.5 ± 0.4; 0.8 (96.0-97.0) |
| Subclade B3.1 2021-2023 (n=46) | 86.4 ± 1.0; 1.3 (81.9-88.8) | 96.4 ± 0.8; 0.6 (93.6-97.9) | 96.1 ± 0.6; 0.7 (93.9-97.2) |
| Subclade B3.2 2018 (n=11) | 85.5 ± 0.9; 0.9 (82.9-88.1) | 96.9 ± 0.7; 0.7 (94.2-98.4) | 97 ± 0.4; 0.7 (96.3-97.9) |
| Subclade B3.2 2016 (n=31) | 85.9 ± 1.5; 1.4 (81-87.8) | 97.2 ± 0.4; 0.3 (94.1-97.7) | 98 ± 0.6; 0.8 (97.0-99.0) |
| Subclade B3 2014 (n=3) | 86.3 ± 1.1; 0.4 (84-89.1) | 95.9 ± 0.9; 0.8 (93.1-97.6) | 95.7 ± 0.2; 1.0 (95.5-95.9) |
| Subclade A2/D1 2021-2023 (n=3) | 95.1 ± 0.8; 1.2 (93.6-97.2) | 87.3 ± 0.8; 1.7 (84.3-88.8) | 87.1 ± 0.8; 1.6 (86.0-88.2) |
| Subclade A2/D1 2018 (n=13) | 94.8 ± 1.0; 1.9 (92.8-96.2) | 88.0 ± 0.6; 2.1 (85.2-89.2) | 88.7 ± 0.5; 0.9 (87.8-89.6) |
| Subclade A2/D1 2016 (n=3) | 94.7 ± 1.0; 2.4 (91.4-97.0) | 88.1 ± 0.5; 0.8 (87.1-89.3) | 87.8 ± 0.4; 0.3 (87.4-88.5) |
| Subclade B2 (n=17) | 83.2 ± 1.5; 0.9 (79.9-87.3) | 92.1 ± 1.6; 0.5 (87.7-95.5) | 91.7 ± 1.7; 2.7 (87.7-95.1) |
| Subclade B1 (n=4) | 85.9 ± 1.8; 2.9 (79.5-88.8) | 95.6 ± 0.8; 1.2 (93.2-97.2) | 95.5 ± 0.6; 0.7 (94.4-96.5) |
| Subclade A2/D2 (n=3) | 91.1 ± 0.7; 1.1 (89.6-92.6) | 88.6 ± 1.1; 2.1 (86.0-90.1) | 88.6 ± 1.0; 2.2 (87.2-89.5) |
| Clade C (n= 4) | 86.2 ± 1.6; 2.3 (81.3-89.1) | 89.5 ± 1.6; 0.8 (82.7-92.6) | 90.3 ± 0.9; 1.8 (89.4-91.8) |
| Fermon | 82.1 ± 0.6; 0.7 (79.4-83.5) | 84.3 ± 1; 1.1 (81.3-86.5) | 84.0 ± 0.1; 0.1 (83.7-84.4) |
